# Dental Calculus as a Potential Early Sign of Oral Functional Decline: A Hypothesis-Generating Case Series

**DOI:** 10.7759/cureus.110841

**Published:** 2026-06-14

**Authors:** Tomomi Ueda

**Affiliations:** 1 Department of Dental Hygiene, Sugimoto Dental Clinic, Kyotanabe, JPN

**Keywords:** bilateral mastication, dental calculus, low tongue posture, mouth breathing, oral functional decline

## Abstract

Dental calculus is generally considered a consequence of inadequate plaque control. However, in clinical practice, calculus is sometimes observed repeatedly at specific intraoral sites despite reasonable oral hygiene. Such site-specific, recurrent calculus may reflect localized salivary stagnation associated with oral functional decline rather than hygiene alone. This hypothesis-generating case series describes three clinical presentations involving four patients and explores this possibility. The three presentations included two pediatric cases with localized lingual calculus on the mandibular anterior teeth despite supervised brushing, one dental technician case showing a plateau-like pattern of calculus re-accumulation followed by apparent stabilization, and one removable partial denture case in which localized calculus on the maxillary right posterior denture decreased progressively over serial recall visits after bilateral chewing had been resumed. Taken together, these observations suggest that recurrent, site-specific dental calculus may, in some patients, reflect altered intraoral conditions associated with oral functional decline, including reduced mastication, low tongue posture, and mouth-breathing-related features. Site-specific dental calculus may therefore serve as a possible early clinical clue to oral functional decline in selected patients, although further studies with objective functional assessment and longitudinal follow-up are needed.

## Introduction

Dental calculus has traditionally been regarded as a consequence of inadequate plaque control and insufficient mechanical cleaning [1,2]. Although this interpretation remains clinically important, some recurrent and site-specific deposition patterns encountered in daily practice are not fully explained by a hygiene-centered framework alone [1,2]. In particular, such cases suggest that local environmental factors, beyond brushing behavior alone, may contribute to this process [1,2].

In the present clinical perspective, oral functional decline refers to a reduction in the coordinated activity of the tongue, lips, cheeks, dentition, and surrounding soft tissues that normally helps maintain physiologic stability within the oral environment. From this viewpoint, mouth breathing may influence dentofacial development and oral function [3,4]. It has also been associated with atypical swallowing patterns [5]. In addition, salivary markers and oral microbial conditions have been reported to differ in adolescents with mouth breathing [6]. Taken together, these findings suggest that altered oral function may create local environments in which salivary flow is restricted and self-cleansing is reduced.

In this report, a functional-flow model is proposed in which oral functional conditions influence the local intraoral flow environment, thereby contributing to salivary stagnation and calcification. Under this hypothesis, repeated calculus deposition at the same site may represent a visible sign of a local environment favoring saliva retention. When tongue posture, buccal soft-tissue behavior, or prosthetic relationships create a stagnation-prone space, saliva may be retained locally, prolonged retention may promote calcification, and calculus may emerge repeatedly at that site. From this perspective, dental calculus is understood not only as a hygiene-related deposit, but also as a possible visible marker of recurrent local salivary stagnation [2].

To examine this hypothesis, three clinical presentations are provided: two pediatric cases that may reflect an initiation phase, a dental technician case that appeared to show a plateau-like stabilization phase, and a removable partial denture case that may reflect a recovery phase after bilateral chewing was resumed. These presentations are offered as a hypothesis-generating case series intended to broaden the conventional interpretation of dental calculus beyond plaque control alone.

## Case presentation

Two pediatric patients receiving daily parental finishing brushing showed marked calculus deposition on the lingual surfaces of the mandibular anterior teeth. Because oral hygiene was consistently supported by parental care, persistent accumulation at this site was considered difficult to explain solely by inadequate cleaning. Importantly, no comparable calculus deposition was observed at other intraoral sites, indicating a clearly site-specific pattern.

Frontal clinical photographs showed the tongue visible between the dental arches at rest, suggesting persistent low tongue posture and a tendency toward mouth breathing. Despite supervised daily brushing, calculus repeatedly deposited on the lingual surfaces of the mandibular anterior teeth. These findings were clinically important because low tongue posture may interfere with salivary flow in the floor-of-mouth region, thereby increasing local stagnation and promoting calcification at this site. Taken together, these conditions may have created a site-specific environment favoring repeated calculus formation despite ongoing mechanical plaque removal.

**Figure 1:**
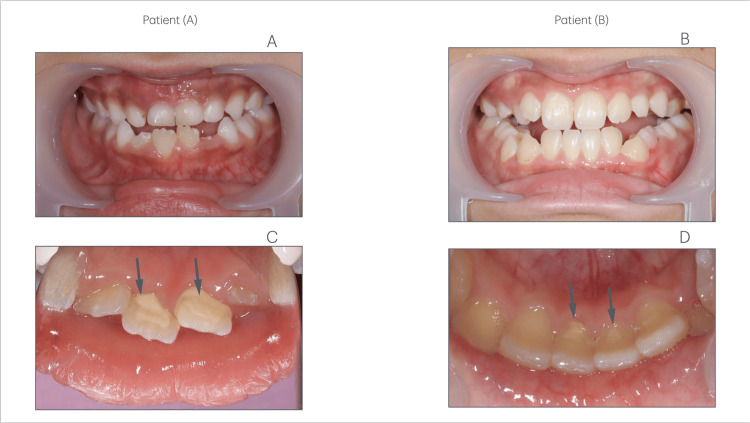
Clinical photographs of pediatric Cases 1A and 1B (A) Frontal intraoral view of pediatric Case 1A. (B) Frontal intraoral view of pediatric Case 1B. (C) Close-up view of the lingual aspect of the mandibular anterior teeth in pediatric Case 1A. (D) Close-up view of the lingual aspect of the mandibular anterior teeth in pediatric Case 1B, showing site-specific calculus deposition. The arrows indicate the areas of calculus deposition.

These pediatric cases were interpreted as representing the initiation phase of the proposed model, suggesting that changes in oral functional conditions may precede and shape the site-specific distribution of calculus. Representative clinical photographs of the two pediatric cases are shown in Figure 1.

A dental technician with professional-level self-care repeatedly developed calculus on the lingual surfaces of the mandibular anterior teeth. In photographs recorded over time after scaling in 2019, calculus gradually re-accumulated and then stabilized instead of continuing to enlarge. At long-term follow-up on January 22, 2026, no clear renewed progressive enlargement was observed compared with the earlier plateau state. However, because the interval period was not observed under a controlled intervention protocol, the 2026 image should be interpreted cautiously as supportive long-term follow-up rather than primary evidence.

This pattern is difficult to explain solely based on oral hygiene. If the deposit reflected persistent cleaning failure alone, more continuous or progressive enlargement would be expected over time. By contrast, in this case, the deposit gradually re-accumulated and then stabilized at a certain level. The 2019 serial observations suggested a plateau-like pattern characterized by gradual re-accumulation followed by apparent stabilization rather than continuous growth. This stabilization, in turn, suggests that a local environment favoring salivary stagnation may have persisted at the affected site, allowing calcification to progress while limiting further increases in total deposition.

**Figure 2:**
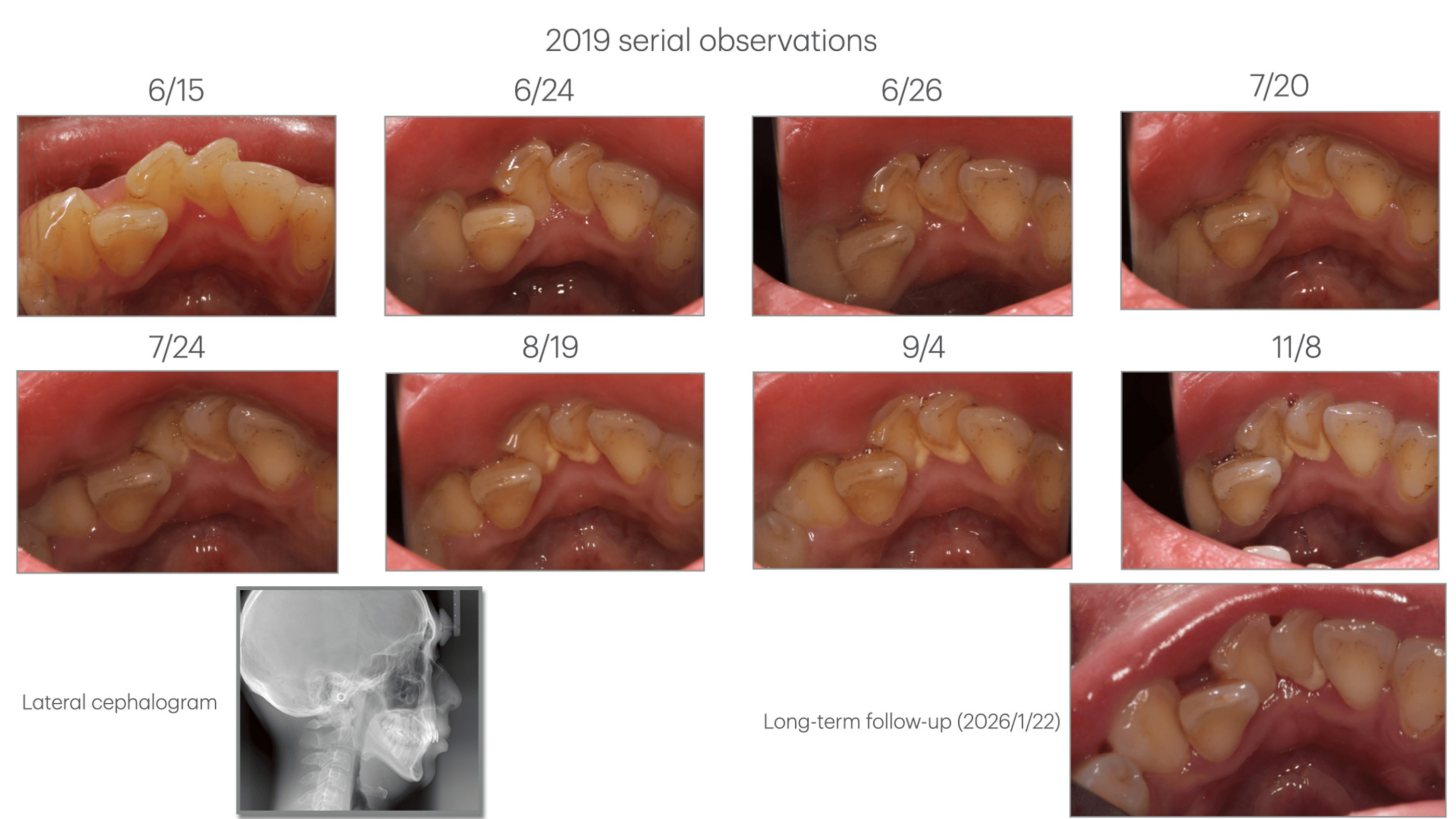
Serial intraoral observations in 2019 and long-term follow-up findings in Case 2 Upper two rows: serial intraoral photographs obtained in 2019, showing gradual re-accumulation of calculus on the lingual surfaces of the mandibular anterior teeth over time after scaling. Lower left: radiographic image obtained prior to the 2019 observation period. Lower right: long-term follow-up intraoral photograph, showing no clear renewed progressive enlargement compared with the earlier plateau-like stage.

From a functional perspective, this patient had a relatively small jaw and a narrow oral space, and a low tongue posture was suggested by cephalometric findings. These findings support the interpretation that repeated calculus formation in this case may have reflected salivary stagnation shaped by oral functional conditions rather than inadequate self-care. The serial intraoral observations from 2019 and the long-term follow-up findings in Case 2 are shown in Figure 2.

A patient wearing a removable partial denture in the maxillary right posterior region showed marked calculus deposition localized to a specific part of the prosthesis. According to the clinical course, pain, swelling, tooth extraction, healing, and the subsequent prosthetic treatment period were followed by a prolonged period during which the affected side was not used for chewing, creating a persistent non-chewing-side environment. Because the denture was removable and cleaned routinely, selective neglect of a single area was considered unlikely.

Clinically, because the affected side had remained non-chewing for a prolonged period, the buccal soft tissue on that side appeared to drape against the tooth and denture surfaces in a shower-curtain-like manner, potentially creating a localized space prone to salivary stagnation. Under this site-specific stagnation environment, calcification may have been repeatedly promoted over relatively short intervals.

The patient was instructed to resume bilateral chewing. At follow-up over serial recall visits, calculus deposition in the previously affected region progressively decreased, and the previously prominent localized accumulation became minimal. This change may reflect not only mechanical removal but also improvement in the local functional environment, in which bilateral chewing gradually reduced cheek-to-surface contact and altered the stagnation pattern that had promoted calcification.

**Figure 3:**
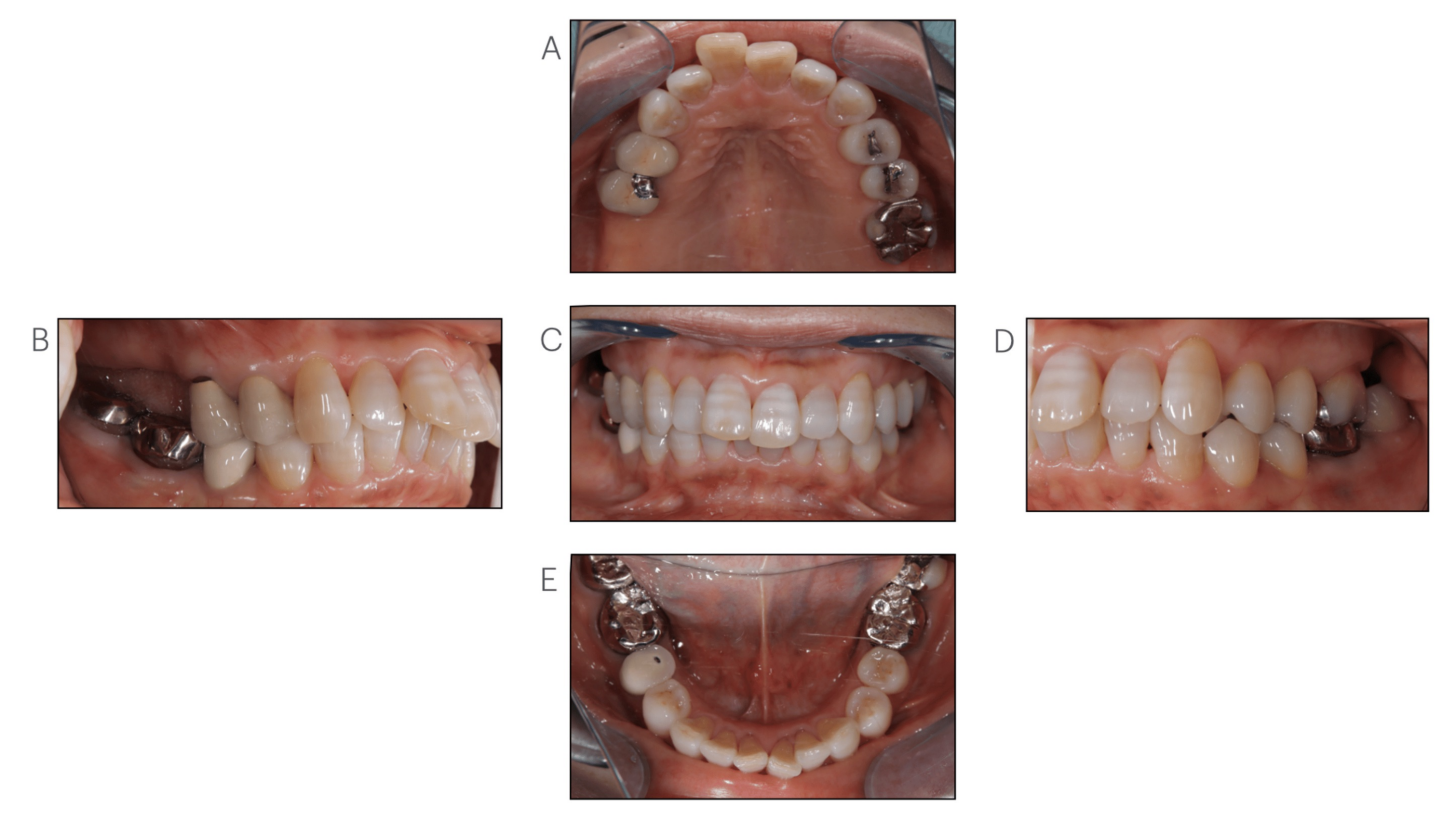
Intraoral status of Case 3 at the initial 2019 presentation (A) Maxillary occlusal view. (B) Right buccal view. (C) Frontal view. (D) Left buccal view. (E) Mandibular occlusal view. The intraoral photographs show the oral condition at the initial 2019 presentation of Case 3, when marked localized calculus was present on the maxillary right posterior denture despite otherwise relatively clean dentition.

This case was interpreted as representing the recovery phase of the proposed model. The progressive reduction of localized calculus after resumption of bilateral chewing suggests that improvement in oral function may reorganize the local salivary flow environment and disrupt stagnation patterns in which calcification had been repeatedly promoted. The intraoral status of Case 3 at the initial 2019 presentation is shown in Figure 3.

**Figure 4:**
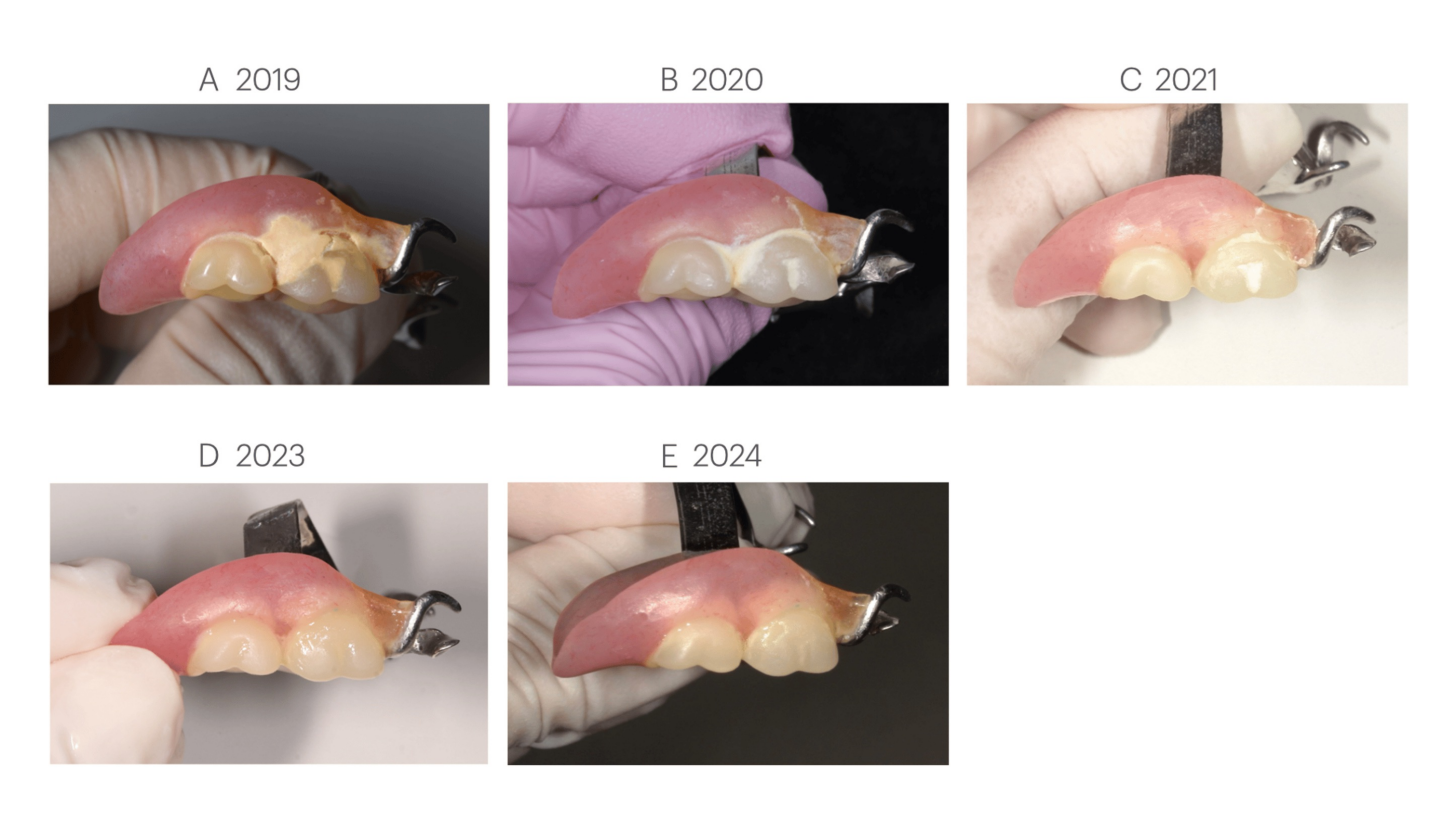
Serial photographs of the removable partial denture in Case 3 from 2019 to 2024 Serial photographs of the same removable partial denture obtained over multiple years, illustrating the long-term clinical course of the prosthesis during the period in which bilateral chewing was reportedly resumed. (A) 2019. (B) 2020. (C) 2021. (D) 2023. (E) 2024.

Serial photographs of the same removable partial denture obtained from 2019 to 2024 are shown in Figure 4.

**Figure 5:**
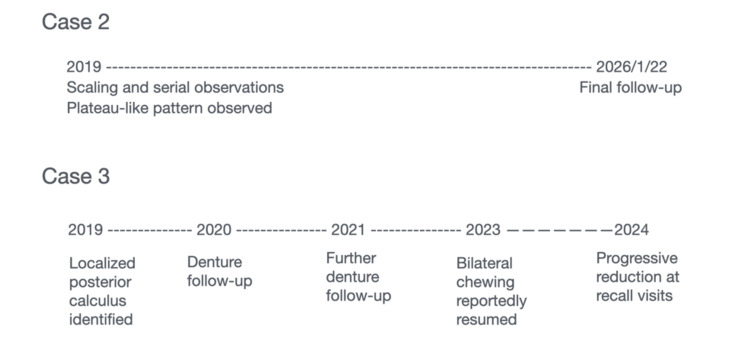
Clinical timeline of longitudinal observations in Cases 2 and 3 The upper timeline summarizes Case 2, including initial scaling in 2019, serial intraoral observations, plateau-like stabilization, and final follow-up on January 22, 2026. The lower timeline summarizes Case 3, including denture-related follow-up from 2019 to 2024, the period in which bilateral chewing was reportedly resumed, and progressive reduction in localized posterior calculus at recall visits. Note: This figure was created by the author using Apple Keynote (Apple Inc., Cupertino, CA, USA).

The longitudinal clinical course of Cases 2 and 3 is summarized in Figure 5.

## Discussion

This case series encourages reconsideration of a hygiene-centered interpretation of dental calculus. In all clinical presentations, calculus recurred at specific sites despite conditions that would ordinarily be expected to improve oral cleanliness, including finishing brushing, professional-level self-care, and routine cleaning of a removable prosthesis [1,2]. These observations do not deny the importance of oral hygiene in calculus formation, but they do suggest that not all recurrent and site-specific deposits can be fully explained by hygiene alone [1,2].

A common feature across these cases is that persistent salivary stagnation may reflect underlying oral functional conditions rather than an isolated local phenomenon. Mouth breathing has been associated with altered dentofacial development and reduced oral function [3,4]. It has also been linked to atypical swallowing patterns [5]. In addition, salivary markers and oral microbial conditions have been reported to differ among adolescents who mouth-breathe [6]. Taken together, these findings suggest that altered oral function may create local environments in which salivary flow is restricted and self-cleansing is reduced.

The plateau-like stabilization observed in Case 2 is informative in this regard. A purely hygiene-based model does not adequately explain why recurrent calculus deposition appears to stabilize at a certain level rather than continuously enlarging. In the present hypothesis, the affected site may function as a stagnation zone shaped by oral function, with a limited spatial capacity. Within such a space, persistent salivary retention may promote calcification and produce a relatively stable deposition pattern over time [2].

Case 3 supports this hypothesis from the opposite direction. The resumption of bilateral chewing was associated with a marked reduction in localized calculus deposition despite no major change in brushing method. This suggests that recovery of function may alter soft-tissue behavior, salivary redistribution, and self-cleansing, thereby improving the local flow environment [4,5]. This interpretation is also consistent with the author’s earlier case report describing a reduction in calculus after functional oral intervention [7], which informed the present hypothesis-generating series.

The comparison between the maxillary posterior buccal region and the mandibular anterior lingual region can also be understood in terms of differing physical conditions rather than simple quantitative differences. In the mandibular anterior lingual region, saliva may be retained in the floor-of-mouth environment, and low tongue posture may prolong retention time and promote calcification [4,6]. By contrast, in the maxillary posterior buccal region, stagnation may occur within a cheek-bounded lateral space under different physical conditions. These functional and physical differences may help explain why similar stagnation-related processes produce different deposition patterns at the two sites [3,4].

**Figure 6:**
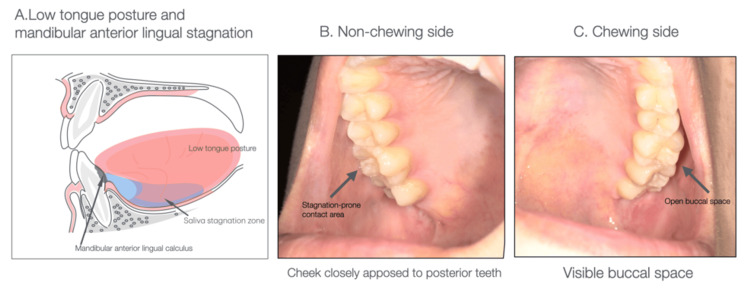
Proposed functional-flow model illustrating anterior lingual stagnation and buccal soft-tissue relationships on the non-chewing and chewing sides (A) Schematic illustration of low tongue posture associated with saliva stagnation along the lingual aspect of the mandibular anterior teeth, promoting localized calculus formation. (B) Clinical photograph of the non-chewing side showing the cheek closely apposed to the posterior teeth, with reduced buccal space. (C) Clinical photograph of the chewing side showing a visible open buccal space adjacent to the posterior teeth. Together, these panels illustrate two site-specific intraoral environments in which altered oral function may contribute to localized salivary stagnation and recurrent calculus formation. Note: Panel A was created by the author using Apple Keynote. Panels B and C are original clinical photographs; arrows and panel labels were added by the author using Apple Keynote (Apple Inc., Cupertino, CA, USA).

The prosthesis case also suggested that cheek apposition may create a localized stagnation-prone space on the maxillary posterior buccal side. This mechanism may be functionally analogous to the stagnation environment on the lingual surfaces of the mandibular anterior teeth, although the physical conditions are not identical. The proposed functional-flow model is summarized in Figure 6. In the mandibular anterior lingual region, saliva may be retained along the floor of the mouth, and low tongue posture may further prolong that retention, thereby promoting calcification. On the maxillary posterior buccal side, local stagnation may arise when the cheek closely apposes the posterior teeth and reduces the buccal space through which saliva from the parotid region would otherwise flow more freely. This difference may help explain why both sites may become prone to localized calculus formation while showing different deposition patterns.

This report has several limitations. It is based on a small number of observational cases and does not include objective quantitative measurements of salivary flow, tongue position, chewing behavior, local flow dynamics, or calculus volume. In addition, oral functional findings were assessed clinically rather than by standardized scoring or instrumental evaluation. Therefore, these findings should be interpreted as clinically grounded, hypothesis-generating observations rather than proof of causality. Nevertheless, the consistent findings of site-specific recurrence, plateau-like stabilization, and reduction following functional recovery warrant further investigation. Future studies combining objective functional assessment, standardized imaging, and longitudinal follow-up are needed to determine whether repeated calculus deposition at the same site can serve as a practical clinical indicator of local oral functional decline and saliva-retaining environments [2-6].

## Conclusions

These observations suggest that repeated calculus deposition at specific sites may reflect not merely inadequate cleaning, but oral functional conditions that promote salivary stagnation. In particular, low tongue posture, altered soft-tissue contact, reduced bilateral mastication, and insufficient chewing may contribute to the formation of stagnation-prone environments in which calcification is facilitated. Although this report is observational and hypothesis-generating, it supports a broader clinical perspective in which persistent recurrent calculus should prompt evaluation not only of plaque control, but also of oral function, soft-tissue dynamics, and the local salivary flow environment. Site-specific dental calculus may serve as a possible early clinical clue to oral functional decline in selected patients.
